# Quality of life and treatment burden of children receiving daily growth hormone treatment in Greece

**DOI:** 10.1007/s12020-025-04269-w

**Published:** 2025-06-09

**Authors:** Athanasios Christoforidis, Fotini-Eleni Karachaliou, Assimina Galli-Tsinopoulou, Dionisios Chrysis, Christina Kanaka-Gantenbein, Evangelia Baxevanidi, Ioannis Skiadas, Oresteia Zisimopoulou, Apostolia Poimenidou, Dimitrios Tsilakis, Elpis-Athina Vlachopapadopoulou, the GHEA Study Group, Georgia Sotiriou, Eleni Tsintzou, Vasiliki-Rengina Tsinopoulou, Alexandra Efthymiadou, Michaela Nikolaou, Sofia Lekka-Emiri

**Affiliations:** 1 First Department of Paediatrics, School of Medicine, Faculty of Health Sciences, Aristotle University of Thessaloniki, Ippokratio General Hospital, Thessaloniki, Greece; 2 Third Department of Paediatrics, Medical School, National and Kapodistrian University of Athens, University General Hospital “ATTIKON”, Athens, Greece; 3 Second Department of Paediatrics, School of Medicine, Faculty of Health Sciences, Aristotle University of Thessaloniki, AHEPA University General Hospital, Thessaloniki, Greece; 4 Division of Paediatric Endocrinology, Department of Paediatrics, Medical School of Patras, University Hospital, Rio, Patras, Greece; 5 Division of Endocrinology, Diabetes and Metabolism, “Aghia Sophia” Children’s Hospital Endo-ERN Center for Rare Paediatric Endocrine Disorders, Athens, Greece; 6 First Department of Paediatrics, Medical School, National and Kapodistrian University of Athens, “Aghia Sophia” Children’s Hospital, Athens, Greece; 7 Pfizer Hellas S.A., Athens, Greece; 8 Department of Endocrinology-Growth and Development, General Children’s Hospital “Panagiotis & Aglaia Kyriakou”, Athens, Greece

**Keywords:** Growth hormone deficiency, Children, Health-related quality of life, Treatment burden, Greece, Growth hormone treatment

## Abstract

**Purpose:**

This study aimed to assess the paediatric growth hormone deficiency burden in Greek patients and their caregivers regarding the health-related quality of life (HRQoL), impact of recombinant human growth hormone (rhGH) treatment, and disease management.

**Method:**

In this cross-sectional study, Quality of Life in Short Stature Youth (QoLISSY) and Life Interference for GHD (LIQ-GHD) questionnaires were administered to both patients with pGHD aged 4–17 years receiving rhGH for minimum 12 months and their caregivers.

**Results:**

This study included 250 patients and their caregivers. The mean ( ± SD) total QoLISSY scores were 80.93 (±14.63) and 76.32 (±17.68) reported by patients and caregivers, respectively. Patients ≥ 8 years scored higher in total and emotional domains compared to caregivers, whereas the treatment domain was lower in patients > 12 years versus caregivers. Ease of injection schedule, life interference, and willingness to continue LIQ-GHD domains mainly comprised treatment burden, with mean (±SD) scores of 19.88 (±22.02), 19.36 (±18.39) and 19.25 (±19.60), respectively. Treatment adherence in the overall population was satisfactory. Patients ≥ 8 years missed more injections than younger individuals.

**Conclusion:**

A good HRQoL and a mild treatment burden were reported by Greek patients receiving daily rhGH and their caregivers; however, variability was observed within children’s and caregivers’ groups. Considering the decreased compliance during adolescence, emphasis should be given to consultations with patients and caregivers to identify issues and address them accordingly. Finally, there is a need to consider alternative treatment regimens which could improve adherence.

## Introduction

Paediatric growth hormone deficiency (pGHD) is a rare and heterogeneous disorder, characterized by inadequate growth hormone (GH) secretion and consequently low insulin-like growth factor-1 (IGF-I), which usually manifests as growth deceleration in children, resulting in short stature and/or growth failure. Since GH has important metabolic effects, such as all lipid and glucose homoeostasis, pGHD can also be associated with metabolic alterations [1, 2]. GHD can be either congenital, acquired, or may even be classified as idiopathic when no clear aetiology can be confirmed [3]. Underlying causes for congenital GHD include genetic defects or anatomical abnormalities [1]. Acquired aetiologies may be the result of pituitary gland insult, including tumors of the sellar region, surgery and radiation, as well as cerebral trauma subsequently of its neuronal control [2]. Even though idiopathic GHD is poorly understood, it appears to be multifactorial [4].

GHD does not only have a physical impact on children, but it is also associated with psychosocial effects and behavioural issues [2], with several studies reporting that children with GHD experience sleep disorders, behavioural problems, lack of self-confidence, anxiety, social withdrawal and depressive symptoms [5, 6]. These may have a significant impact on the health-related quality of life (HRQoL) of patients with GHD, which is considered a crucial factor in treatment decisions. There is evidence demonstrating that short stature is associated with increased burden in physical, emotional and social domains, which all have a direct effect on the HRQoL [7]. Additionally, studies have highlighted that this burden also affects their caregivers [7–11]. However, these findings are not universal and discrepant results were reported from different groups [12].

The therapeutic option for GHD is the recombinant human GH (rhGH) replacement therapy which has a well-established efficacy and safety profile and has been used for more than 30 years for the treatment of children with GHD, as well as for multiple non-GHD growth disorders [13]. Treatment with rhGH has been shown to promote linear growth, improve body composition, maintain euglycemia and normal lipid metabolism, as well as improve the HRQoL, mainly in emotional and social domains [13–15]. Due to the short half-life of current rhGH formulations, daily subcutaneous (SC) injections are required [16]. However, daily injections may pose a burden for both patients and caregivers, as well as a risk for suboptimal treatment adherence [17, 18]. Indeed, a systematic review showed that adherence can vary substantially, reporting a range of 7–71% of children with GHD being non-adherent to the prescribed treatment [17]. Moreover, compliance to rhGH treatment is intrinsically linked to its therapeutic efficacy, while factors predisposing treatment non-adherence are heterogeneous [17, 19–24]. The long duration of rhGH treatment (e.g., ≥5 years) and the challenge of daily administration represent the main reasons for patients’ poor compliance [25, 26]. Considering the lack of data in the Greek setting, the present study aims to assess the burden of GHD on children, adolescents and their caregivers, in terms of HRQoL and impact of the daily rhGH administration, in Greece. In this context, the HRQoL instrument “The Quality of Life in Short Stature Youth” (QoLISSY) questionnaire and the parent–child dyad “Life Interference Questionnaire for GHD” (LIQ-GHD) were used.

## Methods

### Study overview

This cross-sectional study was conducted in 6 Paediatric Endocrinology outpatient clinics in Greece. Data were collected between August 2022 and October 2023.

### Patient population

Eligible patients were ≥ 3 to ≤ 17 years old at consent, with diagnosed idiopathic GHD and who had been under treatment with daily rhGH for at least 12 months before enrolment to the study. Other inclusion criteria were patients’ and caregivers’ sufficient knowledge and understanding of the Greek language. Patients were excluded if they had been receiving drugs other than rhGH with similar route of administration (subcutaneously or intravenously). Patients who were born small for gestational age (SGA), had other underlying aetiologies for rhGH treatment such as patients with Turner’s syndrome, Noonan syndrome, Prader-Willi syndrome, Russell-Silver syndrome, short stature homeobox (SHOX) mutations/deletions, or other chromosomal abnormalities and skeletal dysplasia were excluded. Additional exclusion criteria were undergoing treatment with triptorelin or receiving drug treatment, which, in the investigator’s opinion, might interfere with their ability to understand or answer the questionnaires. Patients participating in interventional trials including those related to long-acting GH, were also excluded.

### Procedures and study assessments

The overall recruiting period was 12 months. A consecutive recruitment approach was recommended to reduce selection bias. Patients were recruited in a single visit and the data collection was completed in the same visit. Two validated questionnaires, the QoLISSY (Quality of Life in Short Stature Youth) and the LIQ-GHD paediatric (Life Interference Questionnaire for Growth Hormone Deficiency) were used in this study and were distributed to the participants in the form of hard copies [27, 28].

The QoLISSY is a condition-specific, HRQoL instrument assessing patients’ (8–18 years) and caregivers’ perspectives [27]. The QoLISSY has been translated to Greek and its psychometric properties have been validated [9, 29]. It includes a total of 50 items for children and adolescents (QoLISSY-Child version, QoLISSY-C) and 66 items for caregivers (QoLISSY-Parent version, QoLISSY-P). It comprises 3 core scales (total of 22 items) which cover the following HRQoL dimensions in the child and caregiver report: Physical, Social, and Emotional. Three additional scales were provided to identify the potential determinants of HRQoL covering coping (10 items), height-related beliefs (4 items) and treatment-specific aspects (14 items). The caregiver-reported version includes 2 additional scales that assess the concerns about the child’s future (5 items) and the effects of short stature on caregivers (11 items) [9, 27]. Questions with a recall period of 1 week are answered on a five-point Likert scale ranging from 1 (never/not at all) to 5 (always/extremely). Values are added within each dimension and transformed to standardized scores (ranging from 1–100) for each individual dimension and for the total HRQoL score, which includes the 3 core domains (physical, social and emotional). Higher values indicate better HRQoL.

The LIQ-GHD paediatric is a Clinical Outcome Assessment (COA) tool, which was developed to evaluate the burden of GH injections in children, adolescents and their caregivers [28]. It is intended for ‘dyad administration’ in which the patient and his/her caregiver read and answer questions together. The LIQ-GHD questionnaire measures 6 hypothesized domains: pen ease of use (PEoU, 5 items), ease of injection schedule (EoIS, 2 items), patient life interference (LI, 5–7 items), satisfaction and willingness to continue treatment (WtC, 2 items), missed injections (2 items) and injection signs and symptoms from patients aged ≥ 8 years (SS, 4 items). Additional domains have been hypothesized for the LIQ-GHD paediatric version: injection signs reported by Caregiver for children aged < 8 years (CS, 2 items), Caregiver Life Interference (CLI, 5–7 items), and family life interference (FLI, 5–6 items). All scores are transformed from raw scores and converted to a 0–100 scale before formal comparisons are made for the endpoint. Even though, the description for the individual items can vary for the same score, a lower score for each endpoint is considered to reflect a better outcome.

### Endpoints

The primary endpoints of this study were: i) to assess the HRQoL of children and adolescents with GHD receiving daily rhGH treatment, as measured by the self-administered questionnaire QoLISSY, and ii) to evaluate patients’ and caregivers’ perception on the burden associated with daily rhGH treatment, as measured by LIQ-GHD. Secondary endpoints included the description of patients’ and caregivers’ demographics and clinical characteristics, the evaluation of caregivers’ burden of disease (through LIQ-GHD) and disease’s psychological impact on caregivers (through QoLISSY). As exploratory objectives, the study, included the identification of possible associations between treatment burden and impact on HRQoL with patients’ age and treatment duration.

### Statistical analyses

Descriptive statistics were used for all primary and secondary endpoints on the full analysis set (FAS), defined as the set that consisted of all participants who signed the informed consent form. Two subpopulations were considered: FAS-Patients and FAS-Caregivers, depending on the analysis output. The strength of linear association between either QoLISSY scores or LIQ-GHD scores with patients’ age (years) and treatment duration (months) were investigated by Pearson’s correlation coefficient. Fisher’s exact test was used to assess the distribution of missed injections (categorized as: 0, 1, 2, ≥3) within the age groups. In addition, age was correlated with the number of missed injections (both treated as numeric variables) by Spearman’s correlation coefficient. Differences between QoLISSY-C and QoLISSY-P were investigated by a Mixed Model for Repeated Measures (MMRM) within patients ≥ 8 years old for each of the instrument domains. Models were based on observed data assuming data are missing at random (MAR assumption). In each model, QoLISSY-C and QoLISSY-P scores were set as the dependent variable. The type of questionnaire (QoLISSY-C, QoLISSY-P) and age groups were used as fixed effects covariates. Patients were randomly fitted, and an unstructured correlation matrix was used. Summaries of the estimated least squared mean differences between QoLISSY-C and QoLISSY-P scores were provided for each age group with the corresponding 95% confidence interval (CI). *P*-values reported were 2-sided and the statistical significance level was set at 0.05. The analysis for generation of the study outputs was performed using R software version 4.2.2.

## Results

### Patient population

The overall study population included 250 children and adolescents (237 aged ≥ 8 years) with idiopathic GHD receiving daily rhGH, and their caregivers. Patient and disease characteristics are presented in Table 1. The peaks of diagnosis were observed at ages 4–6 years and 8–11 years in 30.8 and 39.6% of the cohort, respectively (Supplementary Fig. 1).

**Table 1:** Patient and disease characteristics

	Overall *N* = 250
Parameters	M [ ± SD]
Age (Years)	12.69 [2.90]
Biological sex at birth	n (%)
Male	168 (67.20%)
Female	82 (32.80%)
Height (cm)	146.36 [17.52]
Weight (kg)	42.63 [15.06]
Body Mass Index (kg/m^2^)	19.21 [3.59]
Dose (mg/kg of body weight)^a^	0.02 [0.00]
	n (%)
Thyroxine	53 (21.20%)
Hydrocortisone	3 (1.20%)
Time since diagnosis (Months)	57.91 [31.27]
Time under treatment (Months)	52.95 [31.06]
Time from diagnosis to treatment initiation (Months)	4.96 [7.54]
Age at diagnosis (Years)	7.80 [3.22]
Age at diagnosis (Years)	n (%)
1–3	22 (8.8%)
4–6	77 (30.80%)
7–9	67 (26.8%)
10–12	67 (26.8%)
13–15	17 (6.8%)
Age of treatment initiation (Years)	8.21 [3.21]

*M* mean, *SD* standard deviation

^a^Recombinant human growth hormone

### HRQoL

#### QoLISSY-child and QoLISSY-parent

The QoLISSY-C questionnaire was completed by 237 patients aged 8–17. Moreover, 250 caregivers filled the QoLISSY-P questionnaire (Tables 2 and 3). Caregivers’ demographic characteristics are depicted in Supplementary Table 1.

**Table 2:** QoLISSY-C scores

	Overall *N* = 237		8–12 years *N* = 93		>12 years *N* = 144	
	M	SD	M	SD	M	SD
Physical domain	82.77	14.57	81.50	15.88	83.59	13.66
Social domain	80.02	18.55	80.44	18.57	79.75	18.60
Emotional domain	80.00	16.72	81.82	16.71	78.82	16.67
Coping domain	48.78	21.95	48.76	24.03	48.78	20.58
Beliefs domain	71.28	24.58	71.77	24.51	70.96	24.70
Treatment domain	54.79	20.50	57.18	22.01	53.25	19.38
Total QoLISSY-C score^a^	80.93	14.63	81.25	15.09	80.72	14.38

QoLISSY scores range from 1–100; higher scores denote better health-related quality of life

*QoLISSY-C* QoLISSY-Child, *M* mean, *SD* standard deviation

^a^QoLISSY-C total score is sum of Physical, Social and Emotional domains

**Table 3:** QoLISSY-P scores

	Overall *N* = 250		<8 years *N* = 13		8–12 years *N* = 93		>12 years *N* = 144	
	M	SD	M	SD	M	SD	M	SD
Physical domain	79.38	17.89	65.06	20.24	78.45	18.77	81.28	16.54
Social domain	76.59	20.44	65.14	20.57	77.69	20.17	76.91	20.44
Emotional domain	72.98	19.53	72.12	19.87	77.22	19.48	70.31	19.17
Coping domain	51.62	20.56	40.00	23.36	52.63	22.89	52.01	18.44
Beliefs domain	68.43	25.56	68.27	24.40	70.30	26.46	67.23	25.17
Treatment domain	61.34	20.00	55.36	24.22	60.35	19.84	62.51	19.72
Future Concern domain	76.38	22.86	–	–	–	–	–	–
Effect domain	68.15	21.40	61.89	25.09	70.53	19.85	67.17	21.97
Total QoLISSY-P score^a^	76.32	17.68	67.44	17.82	77.79	17.89	76.17	17.41

QoLISSY scores range from 1–100; higher scores denote better health-related quality of life

*QoLISSY-P* QoLISSY-Parents, *M* mean, *SD* standard deviation

^a^QoLISSY-P total score is sum of Physical, Social and Emotional domains

Both patients and caregivers reported a good HRQoL (scores range from 1–100; higher scores denote better HRQoL), with mean (±SD) QoLISSY total scores (physical/social/emotional subscales) being 80.93 (±14.63) and 76.32 (±17.68), respectively. The highest mean (±SD) score was observed in the physical domain in both groups [82.77 (±14.57) and 79.38 (±17.89) reported by patients and caregivers, respectively], whereas coping was the lowest scored domain, followed by the treatment domain (Tables 2 and 3). Of the QoLISSY-P specific domains, future concern domain received the highest scores [76.38 (±22.86)] and effect domain the lowest scores [68.15 (±21.40)] (Table 3). Nevertheless, the majority of QoLISSY domains scores reported by children (≥8 years old) were higher than those reported by their parents/caregivers, suggesting that children report better HRQoL than perceived by their parents/caregivers. Statistically significant differences were seen for total mean scores between children and caregivers in both age patient subgroups evaluated [8–12 years: Least squares mean (LSM) difference = 3.47, 95% CI: 0.166–6.771, *p* = 0.040; >12 years: LSM difference = 4.55, 95% CI: 1.901–7.209, *p* = 0.001], with caregivers giving lower scores (Supplementary Table 2). Caregivers reported lower scores in the emotional domain items than patients, with the difference being more apparent in patients aged > 12 years (8–12 years: LSM difference = 4.60, 95% CI: 0.740–8.467, *p* = 0.02; >12 years: LSM difference = 8.51, 95% CI: 5.402–11.612, *p* < 0.001). Furthermore, patients reported lower scores than caregivers in the treatment domain, with the difference being statistically significant only for patients > 12 years of age (LSM difference = −9.26, 95% CI: −12.668–−5.859, *p* < 0.001) (Supplementary Table 2).

Correlation assessments between each of the QoLISSY-C domains and age or treatment duration revealed very weak associations of statistical significance in selected cases, in total not supporting noteworthy associations (Supplementary Table 3). Similarly, when QoLISSY-P domain scores were evaluated based on the different age subgroups (<8 years, 8–12 years, ≥12 years) and in relation to treatment duration, very weak associations in selected cases were recorded. Thus, no notable correlations can be claimed (Supplementary Table 4).

#### Treatment burden

A mild treatment burden was reported by patients and their caregivers. The overall mean (±SD) life interference score was 19.36 (±18.39) – scores range from 0–100 and lower scores denote less burden – which was mainly influenced by the fact that patients were bothered by the need for daily injections (Table 4). Among other LIQ-GHD domains, EoIS and WtC mainly comprised treatment burden [mean scores (±SD): 19.88 (±22.02) and 19.25 (±19.60), respectively], whereas PEoU domain received the lowest scores [mean score (±SD): 8.34 (±13.95)]. Regarding the burden related to injections signs and symptoms, a higher score was reported by patients aged ≥ 8 years than caregivers of children aged < 8 years [mean (±SD): 16.91 (±14.66) and 7.31 (±6.96), respectively]. Furthermore, an association was seen between injections signs and symptoms (SS) and age groups in patients ≥ 8 years, with a higher SS burden reported in children aged 8–12 years compared to those > 12 years old [mean (±SD): 20.22 (±15.36) and 14.77 (±13.83) (*p* = 0.005), respectively] (Table 4). However, the correlation between injection signs and symptoms and patients’ age (≥8 years), was considered relatively weak (Pearson’s coefficient: r = −0.187, *p* = 0.004) (Supplementary Table 5). Caregivers reported that daily rhGH injection schedule had low interference in caregiver and family life (daily and social activities, recreation/leisure, spending night away from home and travel), suggesting a reduced treatment burden. Age seemed to be a determinant of the burden in this domain (*p* = 0.021), with FLI subdomain driving this association as higher interference of rhGH injection schedule was reported for families with children aged ≥ 8 years compared to families with younger children (*p* = 0.002) (Table 4). However, further analyses revealed no correlation between FLI scores and age (Supplementary Table 5).

**Table 4:** LIQ-GHD treatment burden scores

	Overall *N* = 250		<8 years *N* = 13		8–12 years *N* = 93		>12 years *N* = 144		*p*-value^a^
	M	SD	M	SD	M	SD	M	SD	
PEoU domain	8.34	13.95	13.08	14.94	9.41	13.93	7.22	13.83	**0.2**
PEoU subdomain for handling	7.53	14.62	12.50	17.68	8.33	14.37	6.55	14.47	**0.3**
PEoU subdomain for overall ease of use	11.60	19.54	15.38	24.02	13.71	19.68	9.90	18.98	**0.3**
EoIS domain	19.88	22.02	25.38	21.06	21.08	22.53	18.61	21.79	**0.5**
LI domain	19.36	18.39	17.86	13.83	20.01	20.59	19.07	17.31	**0.9**
LI with activities subdomain	19.38	19.61	18.08	15.35	19.30	22.48	19.55	18.01	**>0.9**
LI with routine subdomain	18.10	22.40	15.38	19.20	21.24	24.99	16.32	20.76	**0.2**
LI from bothering due to injections	20.50	26.08	19.23	20.80	22.31	27.94	19.44	25.34	**0.7**
WtC domain	19.25	19.60	18.27	14.98	18.41	18.94	19.88	20.45	**0.8**
SS domain	16.91	14.66	–	–	20.22	15.36	14.77	13.83	**0.005**
CS domain	7.31	6.96	7.31	6.96	–	–	–	–	–
CLI/FLI domain	11.87	14.87	9.47	10.81	15.26	17.84	9.90	12.60	**0.021**
CLI subdomain	14.31	17.43	15.11	16.76	17.32	19.24	12.30	16.04	**0.094**
FLI subdomain	9.02	14.15	2.88	4.93	12.86	17.74	7.09	11.28	**0.002**

*LIQ-GHD* life interference questionnaire for growth hormone deficiency, *M* mean, *SD* standard deviation, *PEoU* pen ease of use, *EoIS* ease of injection schedule, *LI* life interference, *WtC* willingness to continue, *SS* injection signs and symptoms reported by patients aged ≥ 8 years, *CS* injection signs reported by caregivers for patients aged < 8 years, *CLI* caregiver life interference, *FLI* family life interferenc3. LIQ-GHD scores range from 0–100; lower scores denote less burden

^a^One-way ANOVA

#### Missed injections

In the overall study population, treatment adherence was relatively good over the four-week period before study initiation, as more than 50% of the participants reported not having missed a single injection (Table 5). Moreover, a proportion of 21.60% declared having missed 1 injection, 14.00% missed 2 injections, and 10.04% more than 3 injections. Missed injections were mostly reported by participants ≥ 8 years of age and the most common reason was “being too tired/just wanting to go to bed”. A significant association was seen between age categories and the distribution of missed injections (categorized as follows: 0, 1, 2, ≥3) (*p* = 0.025) (Table 5). The number of patients who missed ≥ 3 injections was higher amongst adolescents older than 12 years, compared to those in the group of 8–12 and < 8 years of age (14.58 vs. 4.30 vs. 7.69%, respectively). Nevertheless, even though lower adherence rates were observed in children aged ≥ 8 years (53.76 and 52.78% in 8–12 years and > 12 years old, respectively) compared to younger patients (69.23% in < 8 years of age), no noteworthy correlation between age and the number of missed injections was found in further analyses as shown by the very low Spearman’s coefficient value (*r* = 0.132, *p* = 0.038) (Supplementary Table 6). Furthermore, no correlations were appreciated between any of the LIQ-GHD domains with either age or Treatment duration (Supplementary Table 5).

**Table 5:** LIQ-GHD missed injections

	Overall *N* = 250	<8 years *N* = 13	8–12 years *N* = 93	>12 years *N* = 144	*p*-value^a^
**Number of missed injections during the past 4 weeks, Μ (±SD)**	1.08 (2.40)	0.85 (1.52)	0.74 (1.09)	1.32 (2.99)	–
**Number of missed injections during the past 4 weeks, n (%)**					**0.025**
**0**	135 (54.00%)	9 (69.23%)	50 (53.76%)	76 (52.78%)	
**1**	54 (21.60%)	0 (0.00%)	27 (29.03%)	27 (18.75%)	
**2**	35 (14.00%)	3 (23.08%)	12 (12.90%)	20 (13.89%)	
**≥3**	26 (10.40%)	1 (7.69%)	4 (4.30%)	21 (14.58%)	
**Reason for missing injections during the past 4 weeks**^b^**, n (%)**					–
Was too tired/just wanted to go to bed	37 (14.80%)	1 (7.69%)	11 (11.83%)	25 (17.36%)	
Ran out of medication	3 (1.20%)	0 (0.00%)	0 (0.00%)	3 (2.08%)	
Forgot to take the injection	12 (4.80%)	0 (0.00%)	2 (2.15%)	10 (6.94%)	
Was busy with daily activities (such as chores or school)	1 (0.40%)	0 (0.00%)	0 (0.00%)	1 (0.69%)	
Was busy with social activities (such as spending time with family and friends)	2 (0.80%)	0 (0.00%)	0 (0.00%)	2 (1.39%)	
Was busy with recreational/leisure activities (such as playing games, sports, or watching TV)	0 (0.00%)	0 (0.00%)	0 (0.00%)	0 (0.00%)	
Spent the night away from home	8 (3.20%)	0 (0.00%)	2 (2.15%)	6 (4.17%)	
Was travelling/ on vacation	6 (2.40%)	0 (0.00%)	2 (2.15%)	4 (2.78%)	
Was afraid of or refused injection	4 (1.60%)	1 (7.69%)	1 (1.08%)	2 (1.39%)	
Other	2 (0.80%)	0 (0.00%)	0 (0.00%)	2 (1.39%)	

*LIQ-GHD* life interference questionnaire for growth hormone deficiency, *M* mean, *SD* standard deviation

^a^Fisher’s exact test

^b^Categories are not mutually exclusive

## Discussion

### HRQoL

This study evaluated both the HRQoL of Greek children and adolescents receiving daily rhGH and the treatment burden of patients and their caregivers. The present results revealed a good disease-specific HRQoL reported by children and caregivers. Nevertheless, patients reported significantly higher total and emotional mean QoLISSY scores, indicating that children perceived a better HRQoL than caregivers. As stated in previous analyses, a better perception of HRQoL may reflect patients’ tendency to adaptation, especially with rhGH treatment [30], as well as understanding and handling their emotions about GHD better than their parents believe they do. In addition, patients > 12 years of age reported lower treatment domain scores, indicating that their treatment experience may be affected by the fact that they have been receiving therapy for several years. Despite the discrepancies seen between children’s and caregivers’ total QoLISSY scores which were similar to previous reports [30], Drosatou et al. reported weak agreement between QoLISSY-C and QoLISSY-P scores for the coping domain and an overall good agreement for all other domains [9]. Furthermore, the depicted differences seen between patients and caregivers in QoLISSY scores were consistent with other studies evaluating the HRQoL in children and adolescents with chronic health conditions [31].

The current analysis of the data from the QoLISSY questionnaire demonstrated similar scores to the values reported in the QoLISSY User’s Manual [27]. On the one hand, our analysis illustrated similar total QoLISSY-C and QoLISSY-P mean scores to the reference values in the QoLISSY User’s Manual [80.93 (±14.63) vs 79.96 (±18.66) and 76.32 (±17.68) vs 76.98 (±19.41) for QoLISSY-C and QoLISSY-P, respectively] [27]. Similar mean values were also observed for both core and treatment QoLISSY-C domains as well as for QoLISSY-P core domains [27]. On the other hand, both coping and future concern QoLISSY-C scores were slightly lower in our analysis compared to the QoLISSY User’s Manual, [48.78 (±21.95) vs 56.21 (±23.78) and 76.38 (±22.86) vs 83.04 (±21.74), respectively], and slightly higher for both coping and treatment QoLISSY-P domains [51.62 (±20.56) vs 44.14 (±22.85) and 61.34 (±20.00) vs 55.38 (±20.87), respectively] [27]. Other studies using QoLISSY questionnaires in children/adolescents with GHD receiving daily rhGH, reported similar or slightly lower mean total scores than those observed in our analysis [9, 30, 32, 33]. Compared to the present report, a Greek validation study which also used the QoLISSY questionnaire, reported a similar total QoLISSY-C mean score and lower QoLISSY-P scores for all domains, except for the coping related items, where higher scores were observed [9]. The depicted differences could be attributed to the smaller sample of patients included in the Greek validation study. Additionally, the Greek validation study recruited patients from a single centre in Athens, whereas this study collected data from 6 centres in 3 major cities in Greece, thus possibly reflecting a broader perception of HRQoL.

According to Coutant et al, the lowest scores were observed in the coping and treatment domains in both children’s and caregivers’ groups [33], a trend also seen in our analysis. This observation highlights that the short-stature-associated psychological burden remains pivotal for both children and caregivers, indicating that GH treatment experience could be ameliorated. Considering that the current and other studies show that the daily GH treatment affects HRQoL, one could hypothesize that new long-acting GH (LAGH) regimens administered once weekly could contribute to improving the treatment-associated quality of life. Indeed, the study of Loftus et al. which compared the HRQoL of children with GHD receiving daily rhGH vs. LAGH formulations, revealed numerically better HRQoL improvements with LAGH after 12 months of treatment in both QoLISSY-C and QoLISSY-P total and subscale domains in children ≥ 7 years old [30].

### Treatment burden

To our knowledge, the present study is the first to administer the validated dyad LIQ-GHD questionnaire [28] in Greek children and their caregivers. This analysis demonstrated a mild treatment burden which was mainly associated with the rhGH injection schedule, life interference and willingness of patients to continue with treatment, possibly reflecting patients’ difficulties to follow daily administrations. Considering that children with GHD need to receive rhGH treatment for several years, the aforementioned domains are important when choosing a treatment, as they may influence long-term adherence and might subsequently affect clinical outcomes [20, 34, 35]. Moreover, this analysis showed an increased burden related to injection signs and symptoms in younger individuals (8–12 years) than older patients (>12 years), which may reflect an adaptation of older adolescents due to the gradual practice of self-administration.

Nonetheless, the present study revealed that almost 90% of responders missed ≤ 2 injections over a 4-week period, thus, suggesting a good treatment adherence in the overall population. This finding may be explained by the children’s motivation to receive GH treatment to improve their stature, as well as by the function and culture of Greek families. However, our results reported a higher number of missed injections in patients ≥ 8 years of age, indicating adolescents’ challenge to follow daily rhGH regimens over a long period of time. Considering that poor adherence is associated with suboptimal treatment response [20, 21, 34, 35], long-acting regimens have the potential to address adherence issues associated with daily rhGH injections [36–38]. Indeed, a cross-over study that compared the treatment burden in children with GHD receiving daily and weekly GH treatments, reported that the long-acting formulation was associated with lower numbers of missed injections and a higher intension to comply with treatment [36]. In addition, the lower treatment burden, the strong preference for, as well as the greater convenience and satisfaction with weekly administrations [36–38] may contribute to a higher real-world treatment adherence with LAGH and, thus, to improved health-related outcomes.

The LI mean (±SD) score reported in the current analysis, 19.36 (±18.39), was lower than the ones found in similar studies, indicating a milder life interference [33, 36]. Coutant et al, used the LIQ-GHD dyad questionnaire in children aged 3–17 years treated with daily rhGH for at least 4 weeks to assess treatment burden. The results revealed a mean (±SD) total life interference score of 27.7 (±20.7) (*N* = 138) [33]. This agrees with the results reported by Maniatis et al. that also used the LIQ-GHD questionnaire and demonstrated a mean overall LI total score of 24.13 [95% CI (20.61, 27.65) (*N* = 85)] after 12 weeks of daily rhGH treatment in patients aged 3–17 years [36].

Furthermore, adherence rates reported in the current analysis are well in agreement with the existing literature [16, 33]. A systematic review reported mean and median adherence rates ranging from 73.30–95.3% and 91.00–99.20% in 11 and 8 studies, respectively, following daily rhGH administration [16]. An association between age and the distribution of the number of missed injections was detected in the present study. However, our results could not support a correlation between the number of missed injections and patient age, similarly to previous observations on treatment adherence and age [22, 26, 39]. Although Gomez et al. identified adolescence as an adherence barrier in 5 studies [16], an earlier systematic review reported that age was not consistently related to adherence as some studies showed lower adherence with increasing age while others found no association [17]. Several studies have also supported an association between longer treatment duration and lower treatment adherence [16, 20, 21, 24, 33, 40–42].

### Limitations

Nevertheless, our study has several limitations. Since data were collected through questionnaires a recall bias from patients and caregivers cannot be excluded. Given that this was a non-interventional study, a possible selection bias by the investigators needs to be considered. In addition, even though the study was conducted at 6 sites comprising the main referral centers which are located in 3 major Greek cities (Athens, Thessaloniki, and Patra), the risk of underrepresentation of distant locations data should not be ignored.

Furthermore, the limited number of included patients < 8 years of age could pose an issue with subgroup analyses. The limited sample in this subgroup might originate from the fact that less patients are referred for growth deceleration at a younger age, possibly due to parents’ and primary care physicians’ hesitancy. A contributing factor could also be the limited awareness of general paediatricians to refer patients to paediatric endocrinologists. Another restricting factor might be the study’s inclusion criteria which required an ongoing rhGH treatment for the last 12 months. Consequently, while the substantial total number of enrolled children bolsters our conclusions, the unequal distribution across subgroups remains an issue. Similarly to other reports [30, 32, 33], 67% of this study’s participants were males. However, subgroup analysis based on gender was not performed in the present study, thus, the potential sex discordant impact of short stature on both psychological burden and HRQoL was not estimated. Nevertheless, Maghnie et al. demonstrated no significant differences on the psychological burden between genders [32].

## Conclusion

In conclusion, this study demonstrated that children and adolescents with GHD who received daily rhGH treatment for at least 12 months, reported an overall good HRQoL, with children perceiving their HRQoL more positively than their caregivers do. A mild treatment burden was reported, mainly attributed to the ease of injection schedule, the overall life interference, and the willingness to continue the daily rhGH treatment. The treatment adherence was good in the overall study population, with a higher number of missed injections observed in adolescents. Patient and caregiver consultations with the treating team may decrease the daily treatment-associated burden. Finally, this study demonstrated that there is a need to consider alternative treatment regimens for pGHD to improve treatment adherence, especially amongst adolescents.

## Supplementary information

Supplementary Figures

Supplementary Tables

## Data Availability

A subset of data generated or analysed during this study are included in this article and its supplementary material files. The data that support the findings of this study are available from Pfizer Hellas, but restrictions apply to the availability of these data, which were used under license for the current study and so are not publicly available. Further enquiries can be directed to the corresponding author upon reasonable request and with permission of Pfizer.

## References

[1] C.S. Reh, M.E. Geffner, Somatotropin in the treatment of growth hormone deficiency and turner syndrome in pediatric patients: a review. Clin. Pharmacol. **2**(1), 111–122 (2010). 10.2147/CPAA.S6525 22291494 10.2147/CPAA.S6525 PMC3262362

[2] J.G. Halas, A. Grimberg, Dilemmas of growth hormone treatment for GH deficiency and idiopathic short stature: defining, distinguishing, and deciding. Minerva Pediatr. **72**(3), 206–225 (2020). 10.23736/S0026-4946.20.05821-1 32274914 10.23736/S0026-4946.20.05821-1 PMC7490116

[3] M.B. Ranke, Short and long-term effects of growth hormone in children and adolescents with GH deficiency. Front. Endocrinol. **12**, 720419 (2021). 10.3389/fendo.2021.720419 10.3389/fendo.2021.720419 PMC8440916 34539573

[4] A. Grimberg, S.A. DiVall, C. Polychronakos, D.B. Allen, L.E. Cohen, J.B. Quintos, W.C. Rossi, C. Feudtner, M.H. Murad, Guidelines for growth hormone and insulin-like growth factor-I treatment in children and adolescents: growth hormone deficiency, idiopathic short stature, and primary insulin-like growth factor-I deficiency. Horm. Res. Paediatr. **86**(6), 361–397 (2017). 10.1159/000452150 10.1159/000452150 27884013

[5] T. Tanaka, S. Tai, Y. Morisaki, K. Tachibana, Y. Kambayashi, K. Chihara, Y. Seino, K. Fujieda, Evaluation of quality of life in children with GH deficiency and idiopathic short stature using the child behavior checklist. Clin. Pediatr. Endocrinol. **18**(1), 15–22 (2009). 10.1297/cpe.18.15 24790375 10.1297/cpe.18.15 PMC4004879

[6] İ. Akaltun, A. Çayır, T. Kara, H. Ayaydın, Is growth hormone deficiency associated with anxiety disorder and depressive symptoms in children and adolescents?: a case-control study. Growth Horm. IGF Res. **41**(11), 23–27 (2018). 10.1016/j.ghir.2018.06.001 29886327 10.1016/j.ghir.2018.06.001

[7] P. Backeljauw, M. Cappa, W. Kiess, L. Law, C. Cookson, C. Sert, J. Whalen, M.T. Dattani, Impact of short stature on quality of life: a systematic literature review. Growth Horm. IGF Res. **57-58**, 101392–101392 (2021). 10.1016/j.ghir.2021.101392 33975197 10.1016/j.ghir.2021.101392

[8] R. Alsaigh, I. Coyne, Mothers’ experiences of caring for children receiving growth hormone treatment. J. Pediatr. Nurs. **49**, e63–e73 (2019). 10.1016/j.pedn.2019.09.005 31575443 10.1016/j.pedn.2019.09.005

[9] C. Drosatou, E.A. Vlachopapadopoulou, M. Bullinger, J. Quitmann, N. Silva, G. Salemi, I. Pavlopoulou, S. Michalacos, K. Tsoumakas, Validation of the greek version of the quality of life in short stature youth (QoLISSY) questionnaire. J. Pediatr. Endocrinol. Metab. **32**(3), 215–224 (2019). 10.1515/jpem-2018-0403 30735483 10.1515/jpem-2018-0403

[10] K.A. Majewska, M. Stanisławska-Kubiak, K. Wiecheć, M. Naskręcka, A. Kędzia, E. Mojs, Maternal anxiety in relation to growth failure and growth hormone treatment in children. Medicine **99**(37), e22147 (2020). 10.1097/MD.0000000000022147 32925771 10.1097/MD.0000000000022147 PMC7489751

[11] N. Silva, M. Bullinger, R. Sommer, A. Rohenkohl, S. Witt, J. Quitmann, Children’s psychosocial functioning and parents’ quality of life in paediatric short stature: the mediating role of caregiving stress. Clin. Psychol. Psychother. **25**(1), e107–e118 (2018). 10.1002/cpp.2146 28960605 10.1002/cpp.2146

[12] R. Sommer, A. Daubmann, J. Quitmann, U. Ravens-Sieberer, M. Bullinger, Understanding the impact of statural height on health-related quality of life in German adolescents: a population-based analysis. Eur. J. Pediatr. **174**(7), 875–882 (2015). 10.1007/s00431-014-2480-6 25535173 10.1007/s00431-014-2480-6

[13] M.B. Ranke, J.M. Wit, Growth hormone-past, present and future. Nat. Rev. Endocrinol. **14**(5), 285–300 (2018). 10.1038/nrendo.2018.22 29546874 10.1038/nrendo.2018.22

[14] L.G. Gonzalez Briceno, M. Viaud, J. Beltrand, I. Flechtner, Y. Dassa, D. Samara-Boustani, C. Thalassinos, C. Pauwels, K. Busiah, G. Pinto, D. Jaquet, M. Polak, Improved general and height-specific quality of life in children with short stature after 1 year on growth hormone. J. Clin. Endocrinol. Metab. **104**(6), 2103–2111 (2019). 10.1210/JC.2018-02523 30649493 10.1210/jc.2018-02523

[15] J. Quitmann, J. Bloemeke, N. Silva, M. Bullinger, S. Witt, I. Akkurt, D. Dunstheimer, C. Vogel, V. Böttcher, U.K. Krahl, M. Bettendorf, E. Schönau, S. Fricke-Otto, A. Keller, K. Mohnike et al. Quality of life of short-statured children born small for gestational age or idiopathic growth hormone deficiency within one year of growth hormone treatment. Front. Pediatr. **7**, 1–11 (2019). 10.3389/fped.2019.00164 31111024 10.3389/fped.2019.00164 PMC6501464

[16] R. Gomez, S.F. Ahmed, M. Maghnie, D. Li, T. Tanaka, B.S. Miller, Treatment adherence to injectable treatments in pediatric growth hormone deficiency compared with injectable treatments in other chronic pediatric conditions: a systematic literature review. Front. Endocrinol. **13**, 1–11 (2022). 10.3389/fendo.2022.795224 10.3389/fendo.2022.795224 PMC8921265 35299969

[17] S. Graham, J. Weinman, V. Auyeung, Identifying potentially modifiable factors associated with treatment non-adherence in paediatric growth hormone deficiency: a systematic review. Horm. Res. Paediatr. **90**(4), 221–227 (2019). 10.1159/000493211 10.1159/000493211 30522126

[18] M. Brod, L. Højbjerre, S.L. Alolga, J.F. Beck, L. Wilkinson, M.H. Rasmussen, Understanding treatment burden for children treated for growth hormone deficiency. Patient **10**(5), 653–666 (2017). 10.1007/s40271-017-0237-9 28386679 10.1007/s40271-017-0237-9 PMC5605605

[19] B.G. Fisher, C.L. Acerini, Understanding the growth hormone therapy adherence paradigm: a systematic review. Horm. Res. Paediatr. **79**(4), 189–196 (2013). 10.1159/000350251 23635797 10.1159/000350251

[20] S. De Pedro, M. Murillo, I. Salinas, M.L. Granada, M. Martinez, M. Puig-Domingo, A. Andreu, J. Bel, Variability in adherence to rhGH treatment: socioeconomic causes and effect on children’s growth. Growth Horm. IGF Res. **26**, 32–35 (2016). 10.1016/j.ghir.2015.12.002 26774403 10.1016/j.ghir.2015.12.002

[21] F. Bagnasco, N.D. Iorgi, A. Roveda, A. Gallizia, R. Haupt, M. Maghnie, Prevalence and correlates of adherence in children and adolescents treated with growth hormone: a multicenter Italian study. Endocr. Pract. **23**(8), 929–941 (2017). 10.4158/EP171786.OR 28614005 10.4158/EP171786.OR

[22] S. Mohseni, Z. Heydari, M. Qorbani, M. Radfar, Adherence to growth hormone therapy in children and its potential barriers. J. Pediatr. Endocrinol. Metab. **31**(1), 13–20 (2018). 10.1515/jpem-2017-0157 29216008 10.1515/jpem-2017-0157

[23] S. Sultan, M. El-Hourani, É. Rondeau, N. Garnier, Categorizing factors of adherence to parenteral treatment in growth hormone deficiencies and hemophilia: what should be the targets for future research? Patient Prefer. Adherence. **12**, 2039–2063 (2018). 10.2147/PPA.S177624 30349200 10.2147/PPA.S177624 PMC6188171

[24] A. Farfel, S. Shalitin, N. Morag, J. Meyerovitch, Long-term adherence to growth hormone therapy in a large health maintenance organization cohort. Growth Horm. IGF Res. **44**, 1–5 (2019). 10.1016/j.ghir.2018.10.004 30414995 10.1016/j.ghir.2018.10.004

[25] R.G. Rosenfeld, B. Bakker, Compliance and persistence in pediatric and adult patients receiving growth hormone therapy. Endocr. Pract. **14**(2), 143–154 (2008). 10.4158/EP.14.2.143 18308651 10.4158/EP.14.2.143

[26] W.S. Cutfield, J.G.B. Derraik, A.J. Gunn, K. Reid, T. Delany, E. Robinson, P.L. Hofman, Non-compliance with growth hormone treatment in children is common and impairs linear growth. PLoS One **6**(1), 5–7 (2011). 10.1371/journal.pone.0016223 10.1371/journal.pone.0016223 PMC3031542 21305004

[27] The European Qo, L.G., *Quality of Life in Short Stature Youth the QoLISSY Questionnaire User’s Manual* (Pabst Science Publishers, Lengerich, 2013)

[28] D.M. Turner-Bowker, A. Yaworsky, A. Palladino, R.E. Lamoureux, M. Kelly, E. Love, A.M. Pleil, A. Shields, J. Loftus, Development and psychometric evaluation of the life interference questionnaire for growth hormone deficiency (LIQ-GHD) to assess growth hormone injection burden in children and adults. Patient **13**(3), 289–306 (2020). 10.1007/s40271-019-00405-7 31956960 10.1007/s40271-019-00405-7 PMC7210233

[29] J. Bloemeke, N. Silva, M. Bullinger, S. Witt, H.G. Dörr, J. Quitmann, Psychometric properties of the quality of life in short statured youth (QoLISSY) questionnaire within the course of growth hormone treatment. Health Qual. Life Outcomes. **17**(1), 1–15 (2019). 10.1186/s12955-019-1118-9 30885197 10.1186/s12955-019-1118-9 PMC6423839

[30] J. Loftus, J. Quitmann, S.R. Valluri, Health-related quality of life in pre-pubertal children with pediatric growth hormone deficiency: 12-month results from a phase 3 clinical trial of once-weekly somatrogon versus once-daily somatropin. Curr. Med. Res. Opin. **40**(2), 175–184 (2024). 10.1080/03007995.2023.2290623 38053515 10.1080/03007995.2023.2290623

[31] C.A. Hall, C. Donza, S. McGinn, A. Rimmer, S. Skomial, E. Todd, F. Vaccaro, Health-related quality of life in children with chronic illness compared to parents: a systematic review. Pediatr. Phys. Ther. **31**(4), 315–322 (2019). 10.1097/PEP.0000000000000638 31568372 10.1097/PEP.0000000000000638

[32] M. Maghnie, M. Orso, B. Polistena, M. Cappa, G. Pozzobon, D. d’Angela, G. Patti, F. Spandonaro, S. Granato, R. Di Virgilio, D. La Torre, M. Salerno, Quality of life in children and adolescents with growth hormone deficiency and their caregivers: an Italian survey. J. Endocrinol. Invest. **46**(12), 2513–2523 (2023). 10.1007/s40618-023-02106-3 37209402 10.1007/s40618-023-02106-3 PMC10632207

[33] R. Coutant, M. Tauber, B. Demaret, R. Henocque, Y. Brault, F. Montestruc, O. Chassany, M. Polak, Treatment burden, adherence, and quality of life in children with daily GH treatment in France. Endocr. Connect. **12**(4), e220464 (2023). 10.1530/EC-22-0464 36866786 10.1530/EC-22-0464 PMC10083659

[34] J. Loftus, B.S. Miller, C.S. Parzynski, J. Alvir, Y. Chen, P. Jhingran, A. Gupta, M. DeKoven, V. Divino, J. Tse, J. He, M. Wajnrajch, Association of daily growth hormone injection adherence and height among children with growth hormone deficiency. Endocr. Pract. **28**(6), 565–571 (2022). 10.1016/j.eprac.2022.02.013 35263660 10.1016/j.eprac.2022.02.013

[35] J.M. Wit, Achieving optimal short- and long-term responses to paediatric growth hormone therapy. J. Clin. Res. Pediatr. Endocrinol. **11**(4), 329–340 (2019) 31284701 10.4274/jcrpe.galenos.2019.2019.0088 PMC6878339

[36] A.K. Maniatis, M. Carakushansky, S. Galcheva, G. Prakasam, L.A. Fox, A. Dankovcikova, J. Loftus, A.A. Palladino, M. De Los Angeles Resa, C. Turich Taylor, M.T. Dattani, J. Lebl, Treatment burden of weekly somatrogon vs daily somatropin in children with growth hormone deficiency: a randomized study. J. Endocr. Soc. **6**(10), bvac117 (2022). 10.1210/jendso/bvac117 36101713 10.1210/jendso/bvac117 PMC9463876

[37] T. Tanaka, T. Sato, A. Yuasa, T. Akiyama, A. Tawseef, Patient preferences for growth hormone treatment in Japanese children. Pediatr. Int. **63**(10), 1185–1191 (2021). 10.1111/ped.14760 33930225 10.1111/ped.14760 PMC8596999

[38] M. McNamara, D.M. Turner-Bowker, H. Westhead, A. Yaworsky, A. Palladino, H. Gross, A. Pleil, J. Loftus, Factors driving patient preferences for growth hormone deficiency (Ghd) injection regimen and injection device features: a discrete choice experiment. Patient Prefer. Adherence. **14**, 781–793 (2020). 10.2147/PPA.S239196 32431492 10.2147/PPA.S239196 PMC7198440

[39] R.R. Kapoor, S.A. Burke, S.E. Sparrow, I.A. Hughes, D.B. Dunger, K.K. Ong, C.L. Acerini, Monitoring of concordance in growth hormone therapy. Arch. Dis. Child. **93**(2), 147–148 (2008). 10.1136/adc.2006.114249 17768149 10.1136/adc.2006.114249

[40] M.A. Arrabal Vela, C.P. García Gijón, M. Pascual Martin, I. Benet Giménez, V. Áreas del Águila, J.R. Muñoz-Rodríguez, E. Palomo Atance, Adherence to somatotropin treatment administered with an electronic device. Endocrinol. Diabetes Nutr. **65**(6), 314–318 (2018). 10.1016/j.endinu.2018.02.003 10.1016/j.endinu.2018.02.003 29680781

[41] M.C. Maggio, B. Vergara, P. Porcelli, G. Corsello, Improvement of treatment adherence with growth hormone by easypod ™ device: experience of an Italian centre. Ital. J. Pediatr. **44**(1), 113 (2018) 30261918 10.1186/s13052-018-0548-z PMC6161418

[42] N. Lass, T. Reinehr, Low treatment adherence in pubertal children treated with thyroxin or growth hormone. Horm. Res. Paediatr. **84**(4), 240–247 (2015). 10.1159/000437305 26279278 10.1159/000437305

