# Supplementary material for: Quality of life and treatment burden of children receiving daily growth hormone treatment in Greece
Source: Endocrine. 2025 Jun 9;89(3):858–68. doi: 10.1007/s12020-025-04269-w (PMC12370556; doi:10.1007/s12020-025-04269-w)

**SUPPLEMENTARY INFORMATION_FIGURES**

**Quality of life and treatment burden of children receiving daily growth hormone treatment in Greece**

**ENDOCRINE**

Athanasios Christoforidis^1^, Fotini-Eleni Karachaliou^2^, Assimina Galli-Tsinopoulou^3^, Dionisios Chrysis^4^, Christina Kanaka-Gantenbein^5^, Evangelia Baxevanidi^6^, Ioannis Skiadas^6^, Oresteia Zisimopoulou^6^, Apostolia Poimenidou^6^, Dimitrios Tsilakis^6^, Elpis-Athina Vlachopapadopoulou^7^, and the GHEA Study Group

^1^ 1^st^ Department of Paediatrics, School of Medicine, Faculty of Health Sciences, Aristotle University of Thessaloniki, Ippokratio General Hospital, Thessaloniki, Greece

^2^ 3^rd^ Department of Paediatrics, University General Hospital “ATTIKON”, Athens, Greece

^3^ 2^nd^ Department of Paediatrics, School of Medicine, Faculty of Health Sciences, Aristotle University of Thessaloniki, AHEPA University General Hospital, Thessaloniki, Greece

^4^ Department of Paediatrics – Paediatric Endocrinology Unit, Panagia i Voitheia University General Hospital of Patras, Patras, Greece

^5^ Division of Endocrinology, Diabetes and Metabolism and Aghia Sophia Children’s Hospital Endo-ERN Center for rare paediatric endocrine disorders, 1^st^ Department of Paediatrics, Medical School, National and Kapodistrian University of Athens, Aghia Sophia Children’s Hospital, Athens, Greece

^6^ Pfizer Hellas S.A., Athens, Greece

^7^ Department of Endocrinology-Growth and Development, Athens General Children's Hospital P. & A. Kyriakou, Athens, Greece

**Corresponding Author**: Evangelia Baxevanidi, Pfizer Hellas S.A., Athens, Greece**.** E-mail address: [eva.baxevanidi@pfizer.com](mailto:eva.baxevanidi@pfizer.com)

**List of Content**

Supplementary Figure 1: Distribution of age at diagnosis (years) in the overall patient population

**Supplementary Figure 1: Distribution of age at diagnosis (years) in the overall patient population (N=250)**


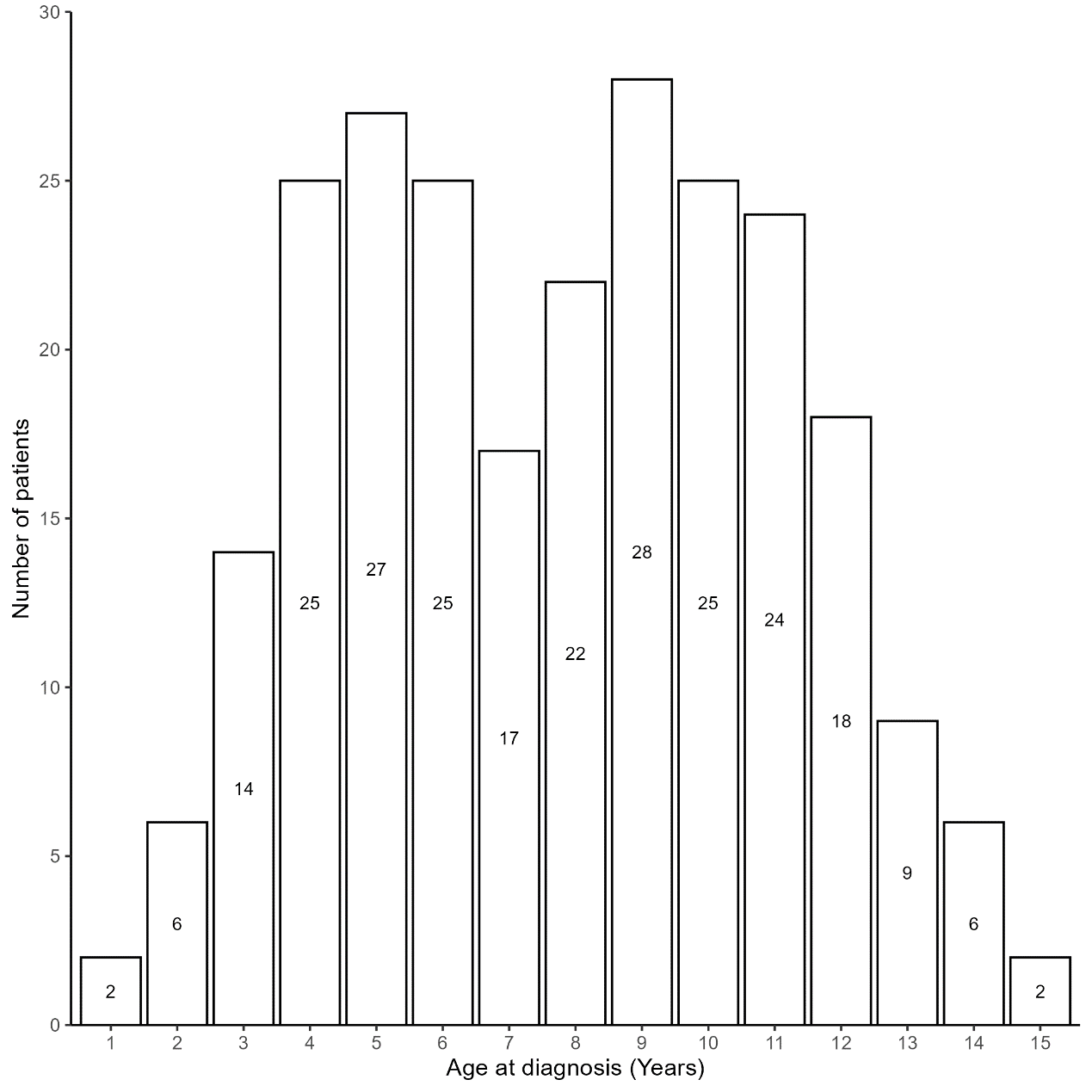

Supplement: Supplementary file 1 — Supplementary Figures [file 12020_2025_4269_MOESM1_ESM.docx]
