# Supplementary material for: Quality of life and treatment burden of children receiving daily growth hormone treatment in Greece
Source: Endocrine. 2025 Jun 9;89(3):858–68. doi: 10.1007/s12020-025-04269-w (PMC12370556; doi:10.1007/s12020-025-04269-w)
Supplement: Supplementary file 2 — Supplementary Tables [file 12020_2025_4269_MOESM2_ESM.docx]

**SUPPLEMENTARY INFORMATION_TABLES**

**Quality of life and treatment burden of children receiving daily growth hormone treatment in Greece**

**ENDOCRINE**

Athanasios Christoforidis^1^, Fotini-Eleni Karachaliou^2^, Assimina Galli-Tsinopoulou^3^, Dionisios Chrysis^4^, Christina Kanaka-Gantenbein^5^, Evangelia Baxevanidi^6^, Ioannis Skiadas^6^, Oresteia Zisimopoulou^6^, Apostolia Poimenidou^6^, Dimitrios Tsilakis^6^, Elpis-Athina Vlachopapadopoulou^7^, and the GHEA Study Group

^1^ 1^st^ Department of Paediatrics, School of Medicine, Faculty of Health Sciences, Aristotle University of Thessaloniki, Ippokratio General Hospital, Thessaloniki, Greece

^2^ 3^rd^ Department of Paediatrics, University General Hospital “ATTIKON”, Athens, Greece

^3^ 2^nd^ Department of Paediatrics, School of Medicine, Faculty of Health Sciences, Aristotle University of Thessaloniki, AHEPA University General Hospital, Thessaloniki, Greece

^4^ Department of Paediatrics – Paediatric Endocrinology Unit, Panagia i Voitheia University General Hospital of Patras, Patras, Greece

^5^ Division of Endocrinology, Diabetes and Metabolism and Aghia Sophia Children’s Hospital Endo-ERN Center for rare paediatric endocrine disorders, 1^st^ Department of Paediatrics, Medical School, National and Kapodistrian University of Athens, Aghia Sophia Children’s Hospital, Athens, Greece

^6^ Pfizer Hellas S.A., Athens, Greece

^7^ Department of Endocrinology-Growth and Development, Athens General Children's Hospital P. & A. Kyriakou, Athens, Greece

**Corresponding Author:** Evangelia Baxevanidi, Pfizer Hellas S.A., Athens, Greece**.** E-mail address: [eva.baxevanidi@pfizer.com](mailto:eva.baxevanidi@pfizer.com)

**List of Content**

Supplementary Table 1: Caregiver demographic characteristics

Supplementary Table 2: Difference between QoLISSY-C and QoLISSY-P within patients ≥8 years old

Supplementary Table 3: Pearson’s correlation between QoLISSY-C Total and individual domain scores with age (years) and Treatment duration (months) within patients ≥8 years old

Supplementary Table 4: Pearson’s correlation between QoLISSY-P Total and individual domain scores with age (years) and Treatment duration (months) within patients ≥8 years old

Supplementary Table 5: Pearson’s correlation between LIQ-GHD Total and individual domain scores with age (years) and Treatment duration (months)

Supplementary Table 6: Spearman’s correlation between LIQ-GHD number of missed injections within patients and age

**Supplementary Table 1:** Caregiver demographic characteristics

| **Parameters** | **Overall**  **N = 250** |
| --- | --- |
| **Age (Years), M [±SD]** | 45.04 [5.72] |
| **Relationship with patient** | **n (%)** |
| **Mother** | 176 (70.40%) |
| **Father** | 74 (29.60%) |
| **Other** | 0 (0.00%) |
| **Caregiver’s level of education** | **n (%)** |
| **ISCED 0 - ISCED 2 (Early childhood to Lower Secondary education)** | 26 (10.40%) |
| **ISCED 3 - ISCED 5 (Upper secondary to Short-cycle tertiary education)** | 112 (44.80%) |
| **ISCED 6: Bachelor’s or equivalent level** | 96 (38.40%) |
| **ISCED 7 - ISCED 8 (Master’s or equivalent level to Doctoral or equivalent level)** | 16 (6.4%) |
| **Work status** | **n (%)** |
| **Full-time employed** | 182 (72.80%) |
| **Part-time employed** | 6 (2.40%) |
| **Part-time employed related to GHD** | 0 (0.00%) |
| **Part-time employed unrelated to GHD** | 6 (100.00%) |
| **Self-employed** | 25 (10.00%) |
| **Retired** | 2 (0.80%) |
| **Student** | 0 (0.00%) |
| **Unemployed** | 11 (4.40%) |
| **Unemployed related to GHD** | 1 (9.09%) |
| **Unemployed unrelated to GHD** | 10 (90.91%) |
| **Engaged on home duties** | 24 (9.60%) |
| **Engaged on home duties related to GHD** | 1 (4.17%) |
| **Engaged on home duties unrelated to GHD** | 23 (95.83%) |
| **On disability or Other** | 0 (0.00%) |
| **Full-time employed (Hrs./week), M [±SD]** | 41.14 [4.51] |
| **Part-time employed (Hrs./week), M [±SD]** | 25.83 [4.92] |
| **Self-employed (Hrs./week)** | 40.64 [4.61] |

M: Mean; SD: Standard deviation

**Supplementary Table 2:** Difference between QoLISSY-C and QoLISSY-P within patients ≥8 years old

|  |  |  | **95% Confidence Interval** | |  |
| --- | --- | --- | --- | --- | --- |
| **Age Group** | **Questionnaire** | **LSM**  **Total Score** | **Lower Bound** | **Upper Bound** | **p-value** |
| **8-12 year** | **QoLISSY-C** | 81.25 | 77.952 | 84.556 | <0.001 |
|  | **QoLISSY-P** | 77.79 | 74.483 | 81.087 | <0.001 |
|  | **(QoLISSY-C) - (QoLISSY-P)** | 3.47 | 0.166 | 6.771 | 0.040 |
| **>12 years** | **QoLISSY-C** | 80.72 | 78.068 | 83.376 | <0.001 |
|  | **QoLISSY-P** | 76.17 | 73.513 | 78.821 | <0.001 |
|  | **(QoLISSY-C) - (QoLISSY-P)** | 4.55 | 1.901 | 7.209 | 0.001 |
|  |  | **LSM Emotional Domain Score** | **Lower Bound** | **Upper Bound** | **p-value** |
| **8-12 year** | **QoLISSY-C** | 81.82 | 78.144 | 85.499 | <0.001 |
|  | **QoLISSY-P** | 77.22 | 73.540 | 80.895 | <0.001 |
|  | **(QoLISSY-C) - (QoLISSY-P)** | 4.60 | 0.740 | 8.467 | 0.02 |
| **>12 years** | **QoLISSY-C** | 78.82 | 75.864 | 81.775 | <0.001 |
|  | **QoLISSY-P** | 70.31 | 67.357 | 73.268 | <0.001 |
|  | **(QoLISSY-C) - (QoLISSY-P)** | 8.51 | 5.402 | 11.612 | <0.001 |
|  |  | **LSM Treatment Domain Score** | **Lower Bound** | **Upper Bound** | **p-value** |
| **8-12 year** | **QoLISSY-C** | 57.18 | 53.081 | 61.281 | <0.001 |
|  | **QoLISSY-P** | 60.35 | 56.250 | 64.449 | <0.001 |
|  | **(QoLISSY-C) - (QoLISSY-P)** | -3.17 | -7.404 | 1.068 | 0.142 |
| **>12 years** | **QoLISSY-C** | 53.25 | 49.954 | 56.544 | <0.001 |
|  | **QoLISSY-P** | 62.51 | 59.218 | 65.807 | <0.001 |
|  | **(QoLISSY-C) - (QoLISSY-P)** | -9.26 | -12.668 | -5.859 | <0.001 |

LSM: Least Squares Mean

Differences between QoLISSY-C and QoLISSY-P were investigated by a Mixed Model for Repeated Measures (MMRM) within patients ≥8 years old. QoLISSY-C and QoLISSY-P scores were set as the dependent variable, whereas type of questionnaire (QoLISSY-C, QoLISSY-P), age groups and their interaction were used as fixed effects covariates.

QoLISSY scores range from 1 to 100; higher scores denote better HRQoL

**Supplementary Table 3:** Pearson’s correlation between QoLISSY-C Total and individual domain scores with age (years) and Treatment duration (months) within patients ≥8 years old

| **Correlation with Age (years)** |  |  |
| --- | --- | --- |
| **QoLISSY-C scores** | **r** | **p-value** |
| **Total** | 0.032 | 0.628 |
| **Physical domain** | 0.132 | 0.042 |
| **Social domain** | 0.016 | 0.808 |
| **Emotional domain** | -0.050 | 0.445 |
| **Coping domain** | 0.078 | 0.231 |
| **Beliefs domain** | 0.014 | 0.828 |
| **Treatment domain** | -0.047 | 0.468 |
| **Correlation with Treatment duration (months)** |  |  |
| **QoLISSY-C scores** | **r** | **p-value** |
| **Total** | 0.212 | 0.001 |
| **Physical domain** | 0.173 | 0.008 |
| **Social domain** | 0.211 | 0.001 |
| **Emotional domain** | 0.172 | 0.008 |
| **Coping domain** | 0.007 | 0.915 |
| **Beliefs domain** | 0.039 | 0.549 |
| **Treatment domain** | -0.013 | 0.841 |

**Supplementary Table 4:** Pearson’s correlation between QoLISSY-P Total and individual domain scores with age (years) and Treatment duration (months) within patients ≥8 years old

| **Correlation with Age (years)** |  |  |
| --- | --- | --- |
| **QoLISSY-P scores** | **r** | **p-value** |
| **Total** | 0.042 | 0.513 |
| **Physical domain** | 0.166 | 0.008 |
| **Social domain** | 0.075 | 0.238 |
| **Emotional domain** | -0.118 | 0.063 |
| **Coping domain** | 0.132 | 0.037 |
| **Beliefs domain** | -0.038 | 0.546 |
| **Treatment domain** | 0.114 | 0.072 |
| **Future Concern domain** | -0.070 | 0.272 |
| **Effect domain** | -0.008 | 0.894 |
| **Correlation with Treatment duration (months)** |  |  |
| **QoLISSY-P scores** | **r** | **p-value** |
| **Total** | 0.160 | 0.011 |
| **Physical domain** | 0.159 | 0.012 |
| **Social domain** | 0.194 | 0.002 |
| **Emotional domain** | 0.086 | 0.175 |
| **Coping domain** | 0.078 | 0.218 |
| **Beliefs domain** | 0.133 | 0.036 |
| **Treatment domain** | 0.136 | 0.031 |
| **Future Concern domain** | 0.074 | 0.245 |
| **Effect domain** | 0.054 | 0.398 |

**Supplementary Table 5:** Pearson’s correlation between LIQ-GHD Total and individual domain scores with age (years) and Treatment duration (months)

| **Correlation with Age (years)** |  |  |
| --- | --- | --- |
| **LIQ-GHD scores** | **r** | **p-value** |
| **PEoU domain** | -0.046 | 0.469 |
| **PEoU subdomain for handling characteristics of injection devices** | -0.040 | 0.524 |
| **PEoU subdomain for overall ease of use the injection pen** | -0.043 | 0.498 |
| **EoIS domain** | -0.041 | 0.520 |
| **LI domain** | 0.068 | 0.284 |
| **LI with activities subdomain** | 0.104 | 0.102 |
| **LI with routine subdomain** | -0.062 | 0.327 |
| **LI from bothering due to injections subdomain** | -0.001 | 0.986 |
| **WtC domain** | 0.075 | 0.238 |
| **SS domain** | -0.187 | 0.004 |
| **CS domain** | -0.299 | 0.321 |
| **CLI/FLI domain** | -0.106 | 0.093 |
| **CLI subdomain** | -0.116 | 0.068 |
| **FLI subdomain** | -0.077 | 0.228 |
| **Correlation with Treatment duration (months)** |  |  |
| **LIQ-GHD scores** | **r** | **p-value** |
| **PEoU domain** | 0.037 | 0.558 |
| **PEoU subdomain for handling characteristics of injection devices** | 0.017 | 0.793 |
| **PEoU subdomain for overall ease of use the injection pen** | 0.083 | 0.191 |
| **EoIS domain** | 0.012 | 0.855 |
| **LI domain** | 0.063 | 0.324 |
| **LI with activities subdomain** | 0.086 | 0.173 |
| **LI with routine subdomain** | -0.008 | 0.905 |
| **LI from bothering due to injections subdomain** | -0.009 | 0.887 |
| **WtC domain** | 0.068 | 0.281 |
| **SS domain** | -0.118 | 0.070 |
| **CS domain** | -0.082 | 0.790 |
| **CLI/FLI domain** | 0.086 | 0.178 |
| **CLI subdomain** | 0.066 | 0.299 |
| **FLI subdomain** | 0.100 | 0.115 |

PEoU: Pen Ease of Use; EoIS: Ease of Injection Schedule; LI: Life Interference; WtC: Willingness to Continue; SS: Injection Signs and Symptoms reported by patients aged ≥8 years; CS: Injection Signs reported by caregivers for patients aged <8 years

**Supplementary Table 6:** Spearman’s correlation between LIQ-GHD number of missed injections within patients and age

| **LIQ-GHD number of missed injections** | **r** | **p-value** |
| --- | --- | --- |
| **Age (years)** | 0.132 | 0.038 |
